# Exploiting ALK inhibition in anaplastic large cell lymphoma: Biological rationale and therapeutic integration

**DOI:** 10.1111/bjh.70458

**Published:** 2026-03-29

**Authors:** Santino Caserta, Enrica Antonia Martino, Mamdouh Skafi, Maria Eugenia Alvaro, Antonella Bruzzese, Nicola Amodio, Marco Fiorillo, Eugenio Lucia, Virginia Olivito, Caterina Labanca, Francesco Mendicino, Ernesto Vigna, Fortunato Morabito, Massimo Gentile

**Affiliations:** ^1^ Hematology Unit, Department of Onco‐Hematology AO of Cosenza Cosenza Italy; ^2^ Emergency and Internal Medicine Department Saint Joseph Hospital East Jerusalem Palestine; ^3^ Department of Experimental and Clinical Medicine University of Catanzaro Catanzaro Italy; ^4^ Department of Pharmacy, Health and Nutritional Science University of Calabria Rende Italy; ^5^ AIL Sezione di Cosenza Cosenza Italy

**Keywords:** ALK inhibitors, ALK‐positive anaplastic large cell lymphoma, precision medicine, relapsed/refractory T‐cell lymphomas, targeted therapy, T‐cell lymphomas, tyrosine kinase inhibitors

## Abstract

Mature T‐cell lymphomas comprise a heterogeneous group of aggressive non‐Hodgkin lymphomas with limited therapeutic options in the relapsed or refractory setting. Among them, anaplastic lymphoma kinase (ALK)‐positive anaplastic large cell lymphoma (ALCL) represents a biologically distinct subtype driven by constitutive activation of ALK fusion proteins, which promote oncogenic signalling through signal transducer and activator of transcription 3, phosphatidylinositol 3‐kinase (PI3K)/AKT Serine/Threonine Kinase 1 (AKT)/mammalian target of rapamycin (mTOR) and mitogen‐activated protein kinase pathways. This molecular dependency provides a strong mechanistic rationale for targeted ALK inhibition. Small‐molecule ALK inhibitors, including crizotinib, have demonstrated high overall response rates (67%–88%) and complete remission rates (~60%–80%) in relapsed or refractory ALK‐positive ALCL, often with rapid clinical responses. Next‐generation ALK inhibitors have shown activity in patients who progress on crizotinib, supporting the concept of sequential ALK‐targeted therapy to overcome acquired resistance. Resistance mechanisms include secondary ALK kinase domain mutations, such as L1196M and G1202R, as well as activation of compensatory signalling pathways, including PI3K/AKT/mTOR, underscoring the importance of molecular reassessment at relapse and the potential role of rational combination strategies. This review critically summarizes the molecular basis of ALK‐driven lymphomagenesis, evaluates the clinical evidence supporting ALK‐targeted therapy and discusses mechanisms of resistance. In addition, it explores emerging strategies for integrating ALK inhibitors into precision‐based management of T‐cell lymphomas, including combination approaches with chemotherapy, immunotherapy or antibody–drug conjugates. Collectively, these developments highlight a paradigm shift towards biology‐driven, personalized therapy in ALK‐positive ALCL.

## INTRODUCTION

Mature T‐cell lymphomas are a heterogeneous group of non‐Hodgkin lymphomas derived from post‐thymic T lymphocytes and include nodal and extranodal entities such as peripheral T‐cell lymphomas (PTCLs), angioimmunoblastic T‐cell lymphoma, anaplastic large cell lymphoma (ALCL), extranodal natural killer (NK)/T‐cell lymphoma, enteropathy‐associated T‐cell lymphoma and hepatosplenic T‐cell lymphoma.^1^ Overall, these neoplasms account for approximately 10%–15% of non‐Hodgkin lymphomas in Western populations, with higher incidence reported in Asia and Latin America.^2^ These malignancies are frequently diagnosed at advanced stages and are associated with poorer long‐term outcomes compared with B‐cell lymphomas. Despite marked biological and clinicopathological heterogeneity among subtypes, many mature T‐cell lymphomas share high rates of treatment failure and disease‐related mortality, representing a persistent clinical challenge.^3^

Brentuximab vedotin (BV), an antibody–drug conjugate that targets CD30, in combination with cyclophosphamide, doxorubicin and prednisone (BV‐CHP), has become the standard first‐line treatment for systemic CD30‐positive ALCL; however, durable remissions are achieved in only a minority of patients. Relapsed or refractory (R/R) disease remains common and carries a particularly unfavourable prognosis, especially among patients who are ineligible for high‐dose chemotherapy followed by autologous stem cell transplantation (ASCT). Historical second‐line approaches, including conventional cytotoxic regimens and BV‐based strategies, have produced median progression‐free survival (PFS) of less than 6–8 months in most series, underscoring a critical unmet need for mechanism‐based therapeutic strategies.^4^

Anaplastic lymphoma kinase (ALK) has emerged as a key oncogenic driver in a biologically distinct subset of mature T‐cell lymphoma, most notably ALK‐positive ALCL. Chromosomal rearrangements involving ALK lead to constitutive kinase activation and aberrant downstream signalling, promoting cellular proliferation, survival and resistance to apoptosis. This non‐redundant oncogenic dependency provides a strong biological rationale for targeted ALK inhibition.^5^

Selective small‐molecule ALK inhibitors have expanded treatment options for ALK‐driven malignancies after BV failure, demonstrating clinically meaningful activity in R/R ALK‐positive ALCL.^6^ This review summarizes the molecular basis of ALK‐driven lymphomagenesis, evaluates clinical evidence for ALK inhibition in the second‐line setting, discusses mechanisms of resistance and safety considerations and outlines future strategies for integrating ALK‐targeted therapies into the evolving management of mature T‐cell lymphomas.

## MOLECULAR BIOLOGY OF ALK‐POSITIVE ALCL

Aberrant ALK activation in T‐cell lymphomas occurs almost exclusively through chromosomal rearrangements that generate constitutively active fusion proteins. The most common event is the t(2;5)(p23;q35) translocation, which produces the Nucleophosmin 1 (NPM1)–ALK fusion protein; however, several alternative fusion partners—including TPM3, TPM4, ATIC and CLTC—have also been described. Despite differences in oligomerization domains and subcellular localization, all ALK fusion proteins retain the intact ALK kinase domain and drive ligand‐independent kinase activation, establishing ALK as the central oncogenic driver in ALK‐positive ALCL.^7^, ^8^

**FIGURE 1:**
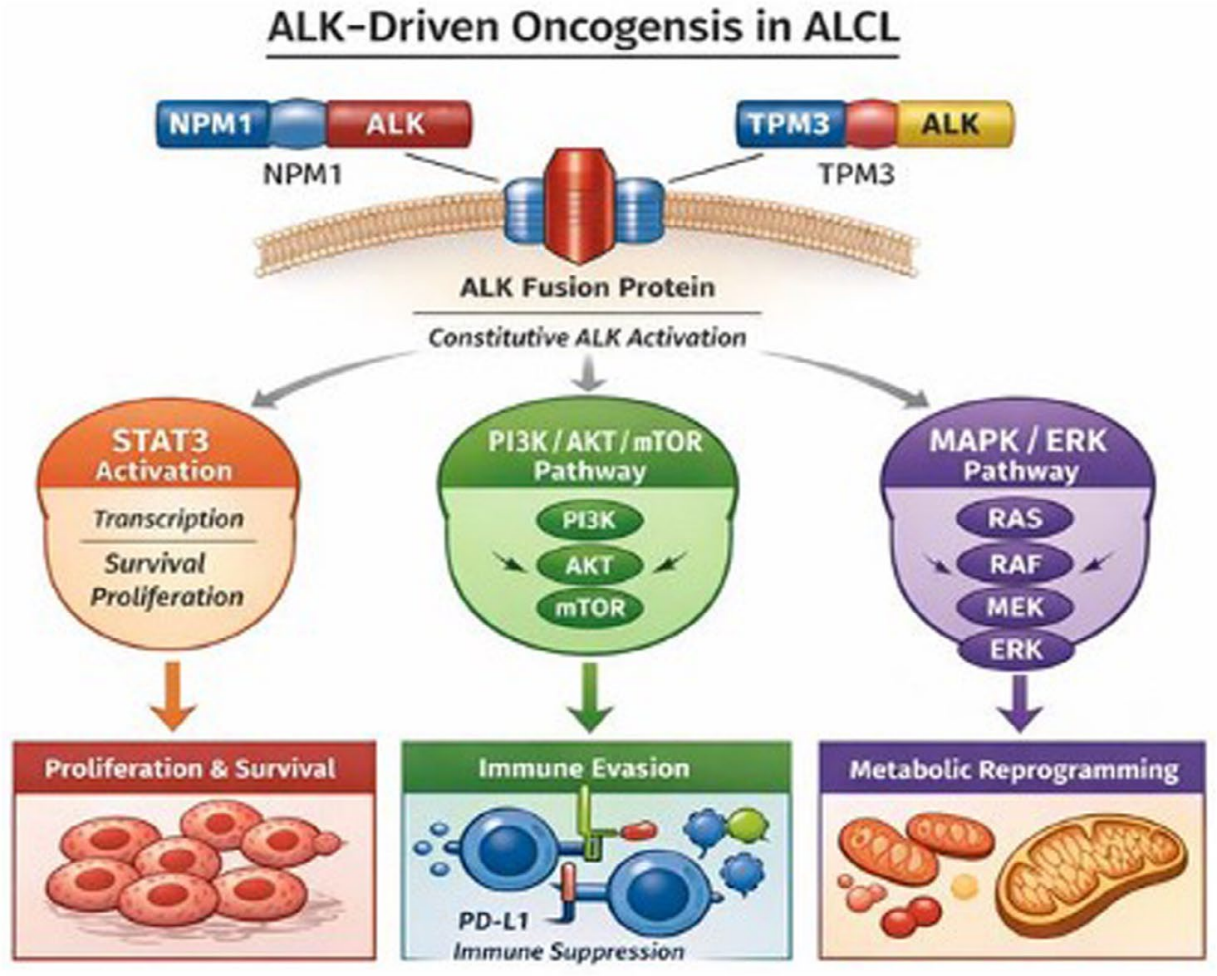
ALK‐driven oncogenic signalling in ALK‐positive T‐cell lymphomas. Schematic representation of common ALK fusion proteins, including the most frequent NPM1–ALK and less common TPM3–ALK fusions, leading to constitutive ALK kinase activation. Constitutive ALK signalling drives three major downstream pathways—STAT3, PI3K/AKT/mTOR and MAPK/ERK—resulting in enhanced proliferation and survival, immune evasion (e.g. via PD‐L1 upregulation) and metabolic reprogramming. These pathways collectively establish a state of oncogene addiction, providing a mechanistic basis for the clinical efficacy of ALK inhibitors in relapsed or refractory ALK‐positive ALCL. This image was created using an artificial intelligence tool (ChatGPT, OpenAI).

Constitutive ALK signalling activates multiple downstream pathways, including signal transducer and activator of transcription 3 (STAT3), phosphatidylinositol 3‐kinase (PI3K)/AKT/mammalian target of rapamycin (mTOR) and mitogen‐activated protein kinase (MAPK)/extracellular signal‐regulated kinase (ERK) cascade. Together, these signalling networks promote cellular proliferation, survival, immune evasion and metabolic reprogramming (Figure 1). Persistent STAT3 activation regulates transcriptional programmes involved in cell‐cycle progression, resistance to apoptosis and immune escape, while activation of the PI3K/AKT/mTOR and MAPK/ERK pathways enhances cellular fitness and contributes to treatment resistance. The integration of these pathways establishes a state of oncogene addiction, rendering AL‐positive tumours highly sensitive to pharmacological ALK inhibition.^9^, ^10^

ALK rearrangements also influence differentiation programmes and epigenetic regulation, shaping the characteristic morphological, immunophenotypic and transcriptional features of ALK‐positive ALCL. This mechanistic dependency provides a robust biological rationale for the clinical efficacy of ALK‐targeted therapies in the R/R setting.

## ALK‐POSITIVE T‐CELL LYMPHOMAS: CLINICAL FEATURES AND OUTCOMES

ALK‐positive ALCL is a biologically and clinicopathologically distinct subset of mature T‐cell lymphomas, predominantly affecting children, adolescents and young adults, although cases can occur across all ages.^11^, ^12^ At diagnosis, patients frequently present with advanced‐stage disease, systemic B symptoms and extranodal involvement, reflecting an aggressive initial clinical phenotype.

Despite this presentation, ALK‐positive ALCL generally demonstrates more favourable outcomes than ALK‐negative disease when treated with standard anthracycline‐based first‐line regimens. High overall response rates (ORRs) and a substantial proportion of durable remissions translate into superior PFS and overall survival (OS).^13^ Nonetheless, R/R disease occurs in a clinically significant subset, underscoring persistent biological and clinical heterogeneity.

Relapses may involve nodal and/or extranodal sites, with some patients experiencing late recurrences after prolonged remission. Prognosis in the R/R setting deteriorates markedly, particularly among patients who fail salvage chemotherapy.^14^ High‐dose chemotherapy followed by ASCT remains a consolidation strategy for chemosensitive patients, while allogeneic transplantation (AlloSCT) may be considered in selected high‐risk cases. However, treatment‐related morbidity and mortality limit its broad applicability.^15^, ^16^

The limited efficacy and cumulative toxicity of conventional salvage regimens underscore the urgent need for biology‐driven approaches. ALK positivity serves as both a predictive and therapeutic biomarker: persistent ALK signalling remains the dominant oncogenic driver after cytotoxic therapy failure,^17^ providing the rationale for incorporating ALK inhibitors into second‐line treatment.^17^

## THERAPEUTIC LANDSCAPE AFTER FIRST‐LINE FAILURE

Conventional salvage chemotherapy, often adapted from aggressive B‐cell lymphoma protocols, has historically formed the backbone of second‐line treatment of R/R T‐cell lymphomas. These approaches yield modest response rates and limited durability, particularly in patients with primary refractory disease or early relapse. Cumulative toxicity, comorbidities and declining performance status frequently restrict the feasibility of intensive chemotherapy in this setting.^18^

High‐dose chemotherapy followed by ASCT is commonly pursued in chemosensitive patients with adequate functional reserve to achieve durable disease control. Nevertheless, a substantial proportion of patients do not reach transplantation due to inadequate salvage response or treatment‐related complications. AlloSCT offers a potentially curative option through a graft‐versus‐lymphoma effect but remains limited to highly selected patients because of significant non‐relapse mortality and long‐term morbidity.^19^

In recent years, targeted agents and immunotherapeutic approaches have expanded the treatment armamentarium in the R/R T‐cell lymphomas, reflecting a shift towards biology‐driven strategies. These include histone deacetylase inhibitors, antifolates, antibody–drug conjugates and immune‐based therapies. Responses are generally heterogeneous and of limited durability, and most agents lack predictive biomarkers, complicating patient selection, sequencing and optimization in routine practice.^20^, ^21^, ^22^, ^23^

Molecularly defined subsets such as ALK‐positive ALCL represent a notable exception. Persistent ALK signalling after failure of cytotoxic therapy provides a compelling biological and clinical rationale for prioritizing ALK‐directed approaches in the second‐line setting. ALK inhibitors are increasingly positioned as mechanism‐based alternatives to conventional salvage chemotherapy, offering higher response rates, reduced toxicity and the potential to serve as a bridge to consolidation strategies when clinically appropriate.

## ALK INHIBITORS: PHARMACOLOGICAL OVERVIEW

ALK inhibitors are small‐molecule tyrosine kinase inhibitors that selectively target the adenosine tiphosphate (ATP)‐binding site of the ALK kinase domain, suppressing aberrant signalling driven by ALK fusion proteins.

Based on chronological development and pharmacological characteristics, ALK inhibitors are classified as first‐, second‐ and third‐generation compounds.^24^

Crizotinib, the first clinically available ALK inhibitor, targets ALK as well as proto‐oncogene, receptor tyrosine kinase (MET) and proto‐oncogene 1, receptor tyrosine kinase (ROS1). In R/R ALK‐positive ALCL, crizotinib demonstrates high ORR (65%–90%) with complete responses (CRs) in approximately 50%–70% of patients across clinical trials and real‐world series. Responses are often rapid and clinically meaningful, although durability may be limited by acquired resistance.^25^ Crizotinib is generally well tolerated, with mostly grade 1–2 adverse events (AEs), including gastrointestinal symptoms, visual disturbances and transient transaminase elevations; grade ≥3 AEs occur in approximately 10%–20% of patients.^26^

Second‐generation inhibitors—including ceritinib, alectinib and brigatinib—were developed to improve potency, pharmacokinetics and activity against resistance mechanisms observed with first‐generation agents. In heavily pretreated patients, these compounds yield an ORR of 60% to >80%, including activity in those previously exposed to crizotinib. CR rates vary from 40% to 70%. Enhanced central nervous system (CNS) penetration, particularly with alectinib, brigatinib and lorlatinib, is an additional pharmacologic advantage, although CNS involvement is less frequent in ALK‐positive ALCL than in other ALK‐driven malignancies. Toxicity profiles differ across agents, reflecting variable off‐target kinase inhibition; treatment discontinuation due to AEs remains relatively uncommon, occurring in fewer than 10%–15% of patients.^27^

Lorlatinib, a third‐generation ALK inhibitor, exhibits potent activity against a broad spectrum of ALK resistance mutations, including compound mutations linked to multidrug resistance. In a small series of R/R ALK‐positive ALCL, lorlatinib achieves response rates exceeding 70%, even in patients who progressed on multiple prior ALK inhibitors. Its toxicity profile includes hyperlipidaemia, neurocognitive effects and weight gain, with grade ≥3 AEs reported in approximately 20%–30% of patients, generally manageable with dose modification and supportive care.^28^

Collectively, ALK inhibitors demonstrate a favourable efficacy–toxicity profile compared with conventional salvage chemotherapy. High ORRs, a substantial proportion of CRs and manageable safety support their integration as a central component of second‐line therapy in ALK‐positive T‐cell lymphomas.

## CLINICAL EVIDENCE OF ALK INHIBITION IN SECOND‐LINE THERAPY

Clinical evidence supporting ALK inhibition in R/R ALK‐positive T‐cell lymphomas is derived primarily from phase I/II trials, expanded access programmes and multicentre retrospective series. Crizotinib is the most extensively studied ALK inhibitor in this setting and has consistently demonstrated robust antitumor activity. In the pivotal Children's Oncology Group phase I/II study, as well as in subsequent expanded access experiences, crizotinib achieved an ORR of 67%–88%, with CR rates of approximately 60%–80%.^29^ Responses were often rapid, including heavily pretreated patients.

Durability of response varies but is clinically meaningful in a substantial proportion of patients. In the French AcSé‐crizotinib trial, the median duration of response exceeded 40 months, with estimated 3‐year PFS and OS of approximately 40% and 60% respectively.^30^ Crizotinib has also been successfully employed as a bridge to consolidation strategies, including ASCT or AlloSCT, particularly in patients achieving CR.

From a safety perspective, crizotinib is generally well tolerated, with mostly grade 1–2 AEs. Grade ≥3 toxicities occur in approximately 20%–35% of patients, whereas treatment discontinuation due to toxicity remains relatively uncommon.^31^, ^32^

Next‐generation ALK inhibitors—including ceritinib, alectinib, brigatinib and lorlatinib—have demonstrated activity in patients who progressed on or were intolerant to crizotinib. Although data are limited to small cohorts and case reports, these agents appear capable of overcoming selected resistance mechanisms.^33^ Lorlatinib, in particular, shows activity in heavily pretreated patients, including those exposed to multiple prior ALK inhibitors, albeit with a higher incidence of grade ≥3 metabolic and neurocognitive AEs (**~**20%–30%).^34^

While randomized comparative trials are lacking, the consistency of efficacy across prospective studies and real‐world series strongly supports the incorporation of ALK inhibitors as a central component of second‐line therapy for this molecularly defined subgroup (Table 1).

**TABLE 1:** Clinical activity and safety of ALK inhibitors in relapsed/refractory ALK‐positive ALCL.

ALK inhibitor	Patient population	ORR (%)	CR (%)	Median DOR	Key grade ≥3 toxicities	Notes
Crizotinib	Paediatric, AYA, adult; relapsed/refractory	67–88	60–80	40+ months (median, AcSé trial)	20%–35%: hepatotoxicity, neutropenia, GI, visual disturbances	Most extensively studied; used as bridge to transplant
Ceritinib	Relapsed/refractory after crizotinib	60–70	40–50	Limited data	GI toxicity, liver enzyme elevations	Small series; overcomes some crizotinib resistance mutations
Alectinib	Relapsed/refractory after crizotinib	65–80	45–60	Limited data	Fatigue, constipation, hepatotoxicity	Improved CNS penetration; small case series
Brigatinib	Relapsed/refractory	60–75	40–50	Limited data	Pulmonary events, elevated CPK	Small series; activity post‐crizotinib
Lorlatinib	Heavily pretreated, including multi‐ALK inhibitor exposure	70+	50+	Limited data	20%–30%: hyperlipidaemia, neurocognitive, weight gain	Active against most ALK resistance mutations; small cohort reports

Abbreviations: AYA, adolescent and young adult; CNS, central nervous system; CPK, creatine phosphokinase; DOR, duration of response; GI, gastrointestinal.

## RESISTANCE TO ALK INHIBITION

Despite the high initial response rates, disease progression due to drug resistance has been increasingly recognized, particularly in patients receiving prolonged therapy.

**FIGURE 2:**
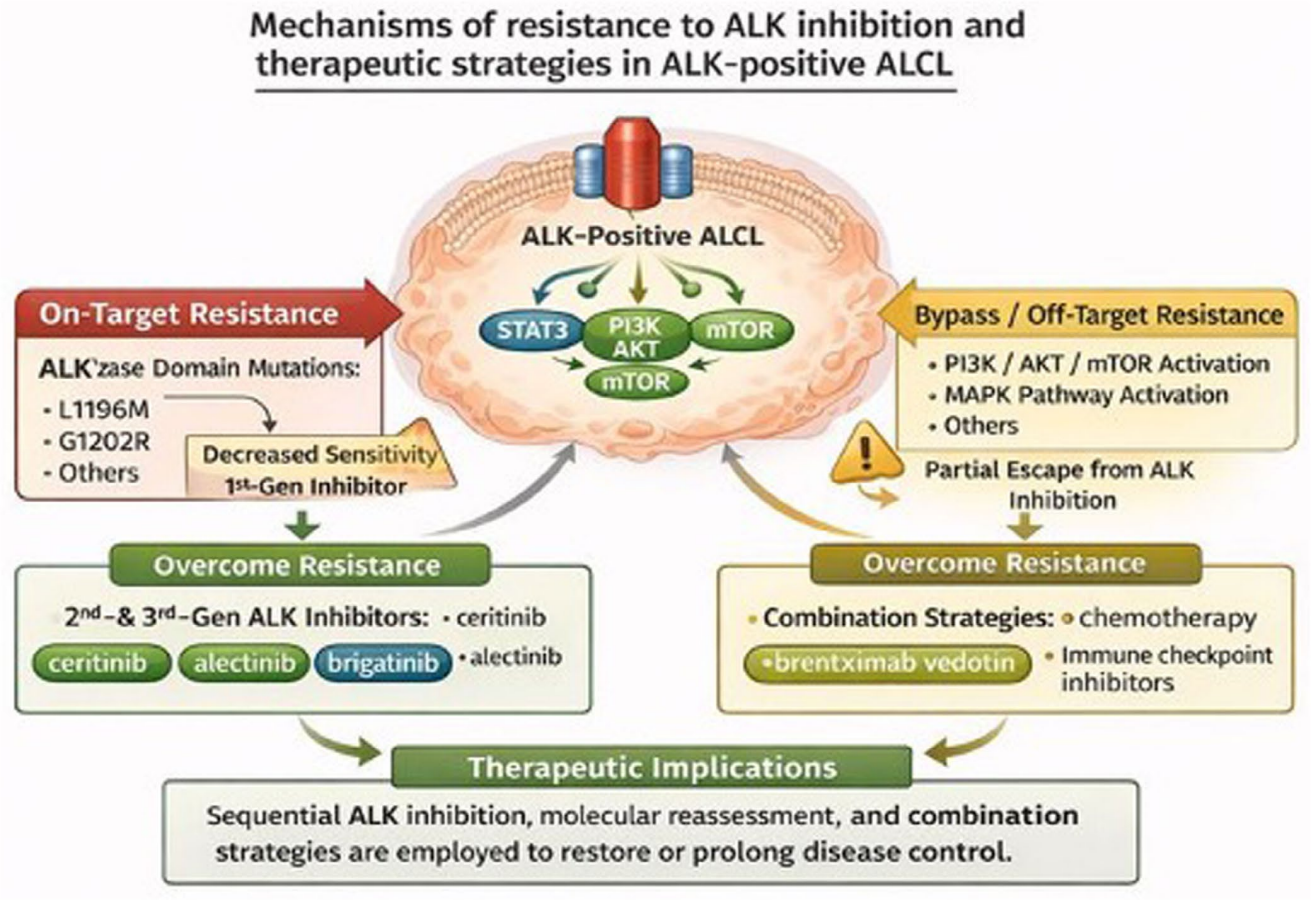
Mechanisms of resistance to ALK inhibition in ALK‐positive anaplastic large cell lymphoma (ALCL) and potential therapeutic strategies. On‐target resistance arises from secondary mutations within the ALK kinase domain (e.g. L1196M, G1202R) that reduce sensitivity to first‐generation inhibitors. Bypass or off‐target resistance involves activation of alternative survival pathways, including PI3K/AKT/mTOR and MAPK/ERK, enabling partial escape from ALK inhibition. Strategies to overcome resistance include the use of second‐ and third‐generation ALK inhibitors with broader mutation coverage as well as combination approaches such as chemotherapy, antibody–drug conjugates (e.g. brentuximab vedotin) and immune checkpoint inhibitors. Sequential ALK inhibition, molecular reassessment at progression and rational combination therapies provide a framework to restore or prolong disease control. This image was created using an artificial intelligence tool (ChatGPT, OpenAI).

Resistance can be classified as primary, defined by lack of initial response, and acquired, emerging after an initial period of disease control. In ALK‐positive ALCL, primary resistance appears uncommon, consistent with the strong oncogenic dependence on ALK signalling, whereas acquired resistance represents the predominant mechanism of treatment failure. The major on‐target and off‐target mechanisms mediating resistance are summarized in Figure 2.

The most frequently implicated mechanism involves secondary mutations in the ALK kinase domain that impair inhibitor binding. Although systematic analyses in ALCL are limited, mutations analogous to those described in other ALK‐driven malignancies, including substitutions affecting the ATP‐binding pocket and activation loop, have been reported in isolated cases. These alterations reduce sensitivity to first‐generation inhibitors such as crizotinib and account for a proportion of relapses following initially durable responses. The incidence of clinically relevant ALK resistance mutations in ALCL appears relatively low.^25^

In addition to on‐target resistance, bypass signalling pathways, including PI3K/AKT/mTOR and MAPK signalling, may sustain tumour cell viability despite effective kinase inhibition. While direct clinical evidence in ALK‐positive ALCL is limited, this mechanism is biologically plausible and supported by translational studies in ALK‐driven malignancies.^30^

Next‐generation ALK inhibitors exhibit increased potency and broader activity against ALK resistance mutations. Lorlatinib, in particular, demonstrates activity against most known ALK kinase domain mutations, including compound mutations associated with resistance to earlier generation inhibitors. Sequential ALK inhibition has restored responses in patients who progressed on crizotinib, supporting the concept of mechanism‐driven therapy sequencing.^35^

Resistance underscores the potential value of molecular reassessment at disease progression to guide therapeutic decisions. Prospective studies incorporating longitudinal molecular analyses will be essential to define the incidence and spectrum of resistance mutations, optimize sequencing strategies and inform rational combination approaches designed to prevent or delay resistance to ALK‐targeted therapy.

## CLINICAL IMPLICATIONS AND THERAPEUTIC ALGORITHMS

The integration of ALK inhibitors into the management of R/R ALK‐positive ALCL represents a paradigm shift from empiric salvage chemotherapy to precision therapy guided by molecular biology. This evolution has important clinical implications for patient selection, choice of agent, timing of intervention and sequencing strategies.

Molecular confirmation at relapse should be standard whenever feasible, as it verifies the persistence of the actionable ALK rearrangement and may reveal clonal evolution relevant to treatment selection. ALK‐positive disease is consistently associated with more favourable outcomes than ALK‐negative counterparts when treated with front‐line anthracycline‐based regimens; however, relapse remains clinically significant and frequently necessitates targeted intervention.^36^

In the second‐line setting, ALK inhibitor selection should prioritize agents with demonstrated clinical activity in ALCL. Crizotinib remains the most extensively studied compound, with ORR approaching 88% substantial CR rates in both prospective and real‐world series. Responses are often durable, with a median duration exceeding 40 months in selected cohorts, and crizotinib can serve both as effective salvage therapy and as an induction platform for consolidation with autologous or allogeneic stem cell transplantation in patients achieving deep remission.^25^, ^29^

Paediatric, adolescent and young adult populations demonstrate particularly favourable outcomes with ALK inhibitors. In a large multi‐institutional retrospective series, targeted therapy (ALK inhibitors or brentuximab vedotin) in relapsed/refractory ALCL was associated with a 5‐year event‐free survival of 63% and a 5‐year overall survival of 91%, highlighting the transformative impact of targeted therapy in this population.^37^

Although ALCL‐specific data on sequential therapy remain limited, insights from ALK‐rearranged NSCLC support the principle of sequential ALK inhibition to prolong disease control. Next‐generation inhibitors—including alectinib, brigatinib, ceritinib and lorlatinib**—**have demonstrated improved PFS and activity against specific resistance mechanisms, including kinase domain mutations and intracranial disease.^38^ Escalation to more potent or broader spectrum ALK inhibitors after crizotinib is biologically and clinically plausible, especially when resistance mutations are documented or the depth of response is suboptimal.

**FIGURE 3:**
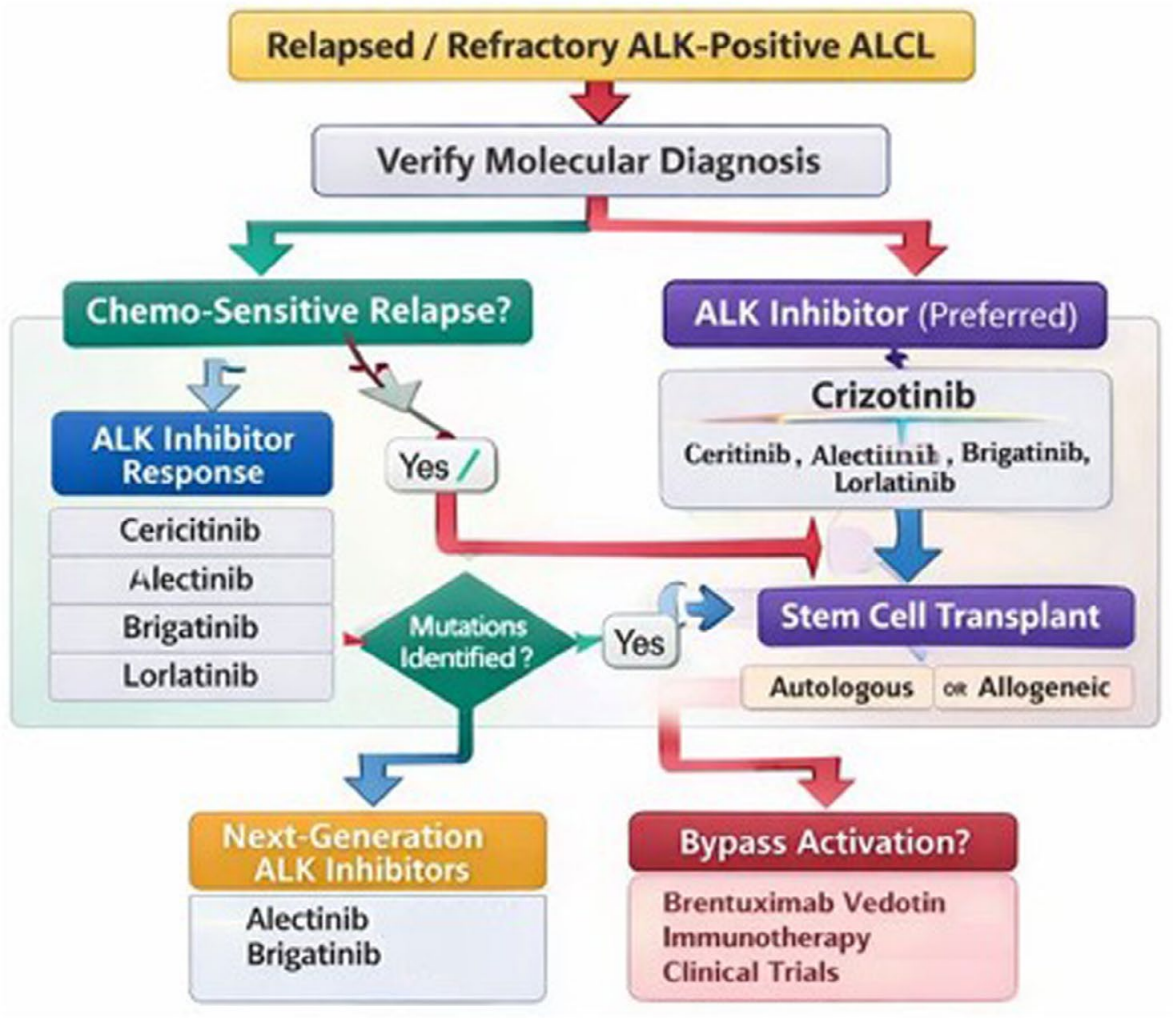
Proposed therapeutic algorithm for relapsed or refractory ALK‐positive anaplastic large cell lymphoma (ALCL). The schematic illustrates a stepwise, biology‐driven approach following first‐line therapy failure. Molecular confirmation of ALK rearrangement at relapse is recommended whenever feasible to guide treatment selection. In the second‐line setting, ALK inhibitors represent the preferred therapeutic option, with crizotinib as the most extensively studied agent and next‐generation inhibitors (ceritinib, alectinib, brigatinib, lorlatinib) considered in cases of intolerance, suboptimal response or acquired resistance. In patients achieving deep remission and with adequate functional reserve, consolidation with autologous or allogeneic stem cell transplantation may be considered. For patients with bypass pathway activation, molecular resistance or limited fitness, alternative strategies including antibody–drug conjugates, immunotherapy or enrolment in clinical trials are appropriate. Treatment decisions should be individualized based on disease biology, response depth, patient characteristics and transplant eligibility. This image was created using an artificial intelligence tool (ChatGPT, OpenAI).

Based on available biological and clinical evidence, Figure 3 summarizes a pragmatic, biology‐driven therapeutic algorithm for R/R ALK‐positive ALCL, positioning ALK inhibitors as the central component of second‐line therapy and integrating molecular reassessment, sequential targeted inhibition and consolidation strategies according to disease behaviour and patient fitness.

ASCT remains context dependent. Adults achieving CR with ALK inhibitors may be considered for autologous transplant to consolidate response, while AlloSCT may be appropriate for high‐risk or multiply relapsed cases, balancing potential curative benefit against treatment‐related morbidity and mortality.^39^

## COMBINATION STRATEGIES AND FUTURE DIRECTIONS

The high response rates observed with single‐agent ALK inhibitors provide a solid rationale for exploring combination strategies designed to deepen responses, extend durability and prevent or delay the emergence of resistance.^25^, ^29^

One approach involves combining ALK inhibitors with conventional chemotherapy. In the Children's Oncology Group trial ANHL12P1, crizotinib added to multi‐agent front‐line chemotherapy in paediatric ALK‐positive ALCL was feasible and yielded high 2‐year event‐free survival. However, an increased incidence of thromboembolic events was observed, emphasizing the need for careful toxicity monitoring when targeted therapy is combined with cytotoxic regimens.^40^ Although conducted in the front‐line setting, this trial establishes proof of principle for chemo–ALK inhibitor combinations that could be relevant in selected R/R scenarios.

Integration with immunotherapeutic strategies represents another promising area of investigation. Preclinical and translational studies suggest that ALK signalling can modulate the tumour immune microenvironment, including regulation of immune checkpoint molecules, such as programmed death‐ligand 1 (PD‐L1). This provides a strong biological rationale for combining ALK inhibition with immune checkpoint blockade. Clinical experience in ALK‐positive ALCL remains limited, but early translational evidence suggests potential synergistic activity, warranting systematic clinical evaluation in future clinical trials.^37^

Targeted agents and antibody–drug conjugates, such as brentuximab vedotin, are additional rational partners for combination or sequential approaches. Sequential or concurrent administration of brentuximab vedotin with ALK inhibitors has been reported in small case series of heavily pretreated patients, resulting in deep and durable remissions in selected cases. These observations provide proof of concept for dual targeting of oncogenic kinases and surface antigens; however, efficacy, optimal scheduling and safety remain to be rigorously defined in prospective studies.^41^

**FIGURE 4:**
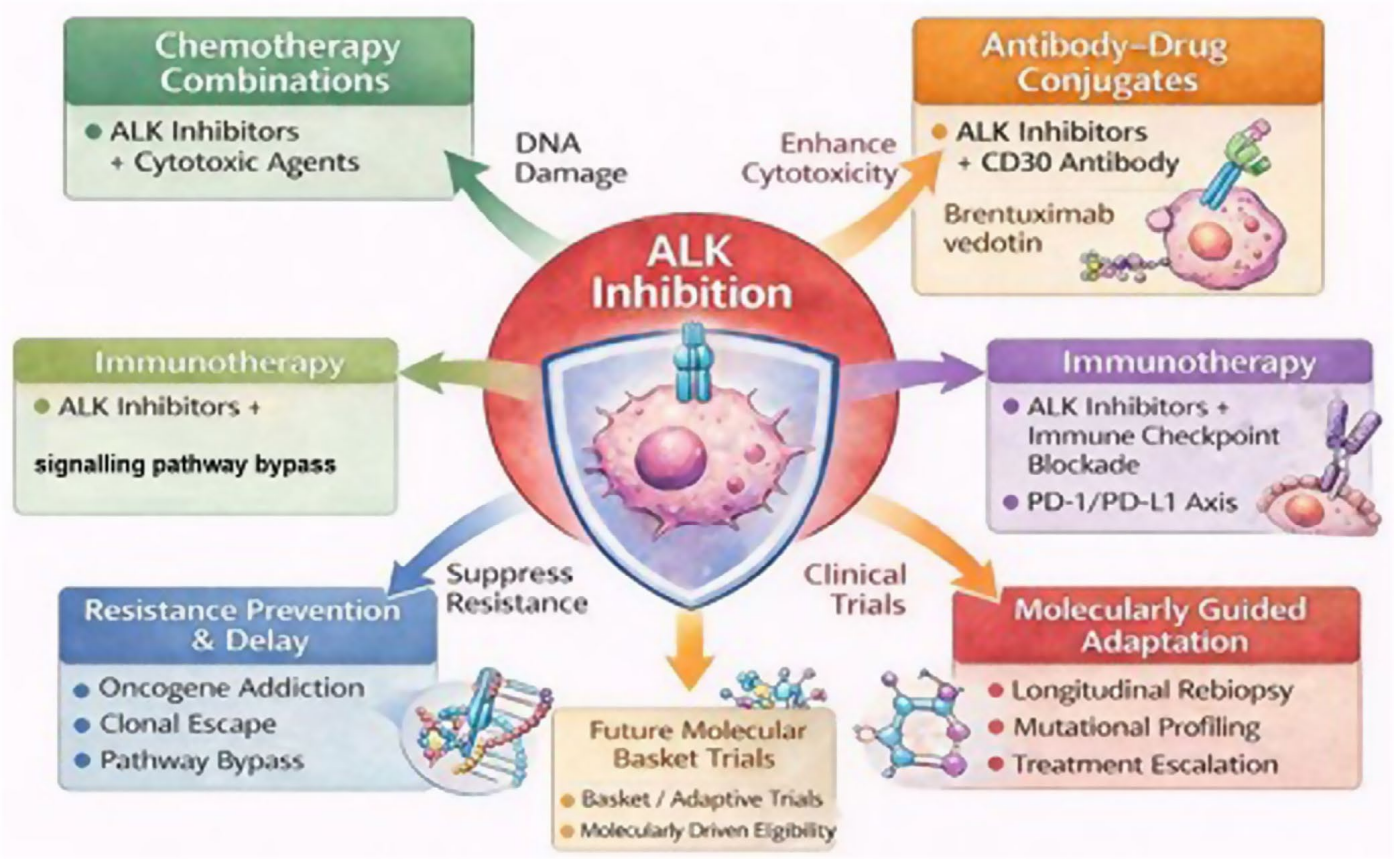
Future directions and rational combination strategies in ALK‐positive ALCL. Schematic representation of potential future strategies to optimize ALK‐targeted therapy. A central ALK inhibition hub is linked to surrounding conceptual elements: Combination approaches including chemotherapy, immunotherapy (ALK inhibitors + cytotoxic agents) and antibody–drug conjugates; resistance prevention and delay strategies; molecularly guided adaptation (longitudinal re‐biopsy, mutational profiling, treatment escalation); and future molecularly guided trials (basket/adaptive design). Arrows indicate conceptual connections rather than sequential treatment steps. This image was created using an artificial intelligence tool (ChatGPT, OpenAI).

Given the rarity of ALK‐positive ALCL, generating robust evidence will require collaborative, multicentre trials and innovative designs, including basket trials based on molecular eligibility rather than histology alone. Collectively, these approaches aim to enhance long‐term disease control and may ultimately redefine curative strategies for this molecularly defined lymphoma subtype (Figure 4).

## CONCLUSIONS

ALK‐positive ALCL represents a distinct molecular and clinical subset of T‐cell lymphomas, predominantly affecting children, adolescents and young adults, and characterized by constitutive activation of ALK kinase due to chromosomal rearrangements. This drives malignant transformation through sustained proliferative signalling, enhanced survival, immune evasion and metabolic reprogramming, establishing a state of oncogene addiction that renders tumour cells highly sensitive to ALK inhibition.^2^

Clinically, ALK‐positive ALCL exhibits relative chemosensitivity, yet relapsed or refractory disease remains a significant challenge, particularly in patients ineligible for ASCT. Conventional salvage regimens are associated with modest efficacy and substantial toxicity, highlighting the unmet need for mechanism‐driven therapies.^5^

ALK inhibitors have transformed the therapeutic landscape in this setting. First‐generation inhibitors, such as crizotinib, achieve high response rates, rapid onset of activity and substantial complete remission rates. Next‐generation inhibitors expand this paradigm, offering increased potency, activity against resistance mutations and improving CNS penetration, although disease‐specific data remain limited due to the rarity of the ALCL.^20^, ^24^ Across studies, ALK inhibitors demonstrate a favourable efficacy–toxicity profile, enabling prolonged administration and use as a bridge to consolidation strategies such as autologous or allogeneic transplantation.

Resistance to ALK inhibition remains a key clinical challenge, most commonly arising from acquired secondary mutations within the ALK kinase domain, with less frequent contributions from bypass signalling pathways, such as PI3K/AKT/mTOR and MAPK/ERK. While primary resistance is uncommon, acquired resistance underscores the importance of molecular reassessment at relapse, sequential use of next‐generation ALK inhibitors and exploration of rational combination strategies to prolong disease control.^30^

Emerging evidence supports combination approaches to enhance the depth and durability of response, including chemo–ALK inhibitor regimens, integration with immunotherapy and dual targeting with antibody–drug conjugates such as brentuximab vedotin. Translational studies indicate that ALK signalling modulates immune checkpoint expression, providing a mechanistic rationale for combining targeted inhibition with immune‐modulating therapies. Early clinical experience suggests feasibility, but robust prospective evaluation through multicentre and molecularly defined basket trials is required.^29^

Optimal clinical management of ALK‐positive ALCL requires an individualized biology‐driven approach accounting for patient age, performance status, prior therapies, depth and duration of remission and transplant eligibility. ALK inhibitors should be prioritized in the second‐line setting, with careful sequencing and consideration of consolidation in responsive patients.^36^ Future research should focus on lymphoma‐specific prospective studies, earlier integration of ALK‐targeted therapy, adaptive strategies guided on molecular profiling and systematic evaluation of combination approaches to prevent resistance and maximize long‐term survival. These efforts will be essential to refine precision‐based treatment paradigms and improve outcomes in this rare but highly targetable lymphoma.

## AUTHOR CONTRIBUTIONS

Santino Caserta, Enrica Antonia Martino, Mamdouh Skafi, Fortunato Morabito and Massimo Gentile: Conceptualization. Enrica Antonia Martino, Francesco Mendicino, Ernesto Vigna, Antonella Bruzzese and Fortunato Morabito: Methodology. Enrica Antonia Martino, Santino Caserta, Fortunato Morabito, Massimo Gentile, Marco Fiorillo and Nicola Amodio: Writing—original draft preparation. Enrica Antonia Martino, Santino Caserta, Mamdouh Skafi, Fortunato Morabito and Massimo Gentile: Writing, review and editing. All authors have read and agreed to the published version of the manuscript.

## FUNDING INFORMATION

The authors have nothing to report.

## CONFLICT OF INTEREST STATEMENT

The authors declare that the research was conducted without any commercial or financial relationships that could be construed as a potential conflict of interest.

## Data Availability

Data sharing does not apply to this article as no new data were generated or analysed in this study.
